# A Water-Soluble Extract from *Actinidia arguta* Ameliorates Psoriasis-Like Skin Inflammation in Mice by Inhibition of Neutrophil Infiltration

**DOI:** 10.3390/nu10101399

**Published:** 2018-10-02

**Authors:** Hyun-keun Kim, Min Jung Bae, Seonung Lim, Wonwoo Lee, Sunyoung Kim

**Affiliations:** 1 School of Biological Sciences, Seoul National University, Seoul 151-742, Korea; yamanin403@snu.ac.kr (H.-k.K.); kindman10@snu.ac.kr (S.L.); 2 ViroMed Co. Ltd., Building 203, Seoul National University, Seoul 151-742, Korea; mjbae1231@viromed.co.kr (M.J.B.); wwlee@viromed.co.kr (W.L.)

**Keywords:** *Actinidia arguta*, PG102, psoriasis, keratinocyte, neutrophil

## Abstract

Psoriasis is a chronic inflammatory disease with complex etiology involving multiple factors. Current treatment methods are highly limited and there is a strong need for the development of safer and efficacious agents. We have previously shown that a water-soluble extract derived from hardy kiwifruit *Actinidia arguta*, called PG102, shows potent anti-inflammatory effects. Based on its reported biological activities, the effects of PG102 were examined on imiquimod-induced psoriasis-like skin inflammation. Our results showed that topical application of PG102 ameliorates clinical symptoms of psoriasis, reducing skin thickness and Interleukin (IL)-17A level in draining lymph nodes without causing any adverse effects. Treatment with PG102 on cytokine-stimulated HaCaT cells suppressed hyperproliferation and downregulated the expression of various chemokines and antimicrobial peptides known to induce neutrophil infiltration. These anti-inflammatory activities of PG102 were mediated via inhibition of NF-κB and signal transducer of activation (STAT) signaling. We also found decreased neutrophil chemotaxis both in vitro and in vivo. Taken together, PG102 has potential as a safe and effective reagent for the treatment of psoriasis.

## 1. Introduction

Psoriasis is a chronic inflammatory skin disease characterized by red and scaly plaques with itching and burning sensations [1]. Psoriatic lesions show distinct histopathological features such as epidermal acanthosis, parakeratosis, and hyperkeratosis [2]. While psoriasis mainly affects skin, it is also associated with various comorbidities: Psoriatic arthritis, metabolic syndromes and cardiovascular diseases [3]. These conditions together substantially impact the quality of life of patients along with placing them under socioeconomic and psychological burdens [4]. Psoriasis affects at least 125 million people globally, but there is no effective treatment method at present [5].

The cross-talk between innate and adaptive immunity has been regarded as a central axis in the pathogenesis of psoriasis, and it is now widely accepted that self-DNA released from the host cell forms complexes with antimicrobial peptide LL-37 produced from keratinocytes [6]. This response stimulates plasmacytoid dendritic cells, and subsequently leads to the production of interleukin 23 (IL-23) by dermal dendritic cells and differentiation of T helper cell 17 (Th17), which is another central axis of psoriasis [7]. In addition to LL-37, other antimicrobial peptides such as β-defensin 2 or S100A8/9 are also known to be overexpressed in psoriatic epidermis, and promote chemotaxis of neutrophils [8,9]. Indeed, neutrophils are one of the most abundant cell types found in psoriatic lesions. They produce various inflammatory mediators including IL-17, and form neutrophil extracellular traps (NETs), exacerbating psoriasis [10].

Topical glucocorticoids are the mainstay of treatment for psoriasis but they cause various side effects from long-term use [11]. Recent advances in the understanding of psoriasis pathobiology have resulted in the development of high-specificity biologics which have shown positive therapeutic effects. However, they have been associated with an increased risk of opportunistic infections and immunogenicity, not to mention the high costs [12]. Since psoriasis is a chronic and recurring disease, safer, and still effective therapeutics that can be used for long-term are in need.

Compounds and extracts from plant sources have often been used to manage chronic inflammatory diseases, in place of conventional immunosuppressors as their safety profiles are well-established. PG102 is a water-soluble extract of hardy kiwifruit *Actinidia arguta,* which is rich in flavonoids such as isoquercitrin, vitamin C, and organic acids such as quinic acid [13,14]. It has been shown to contain strong anti-inflammatory and anti-oxidative activities, alleviating symptoms of spontaneous dermatitis, and atopic dermatitis in respective animal models by regulating the expression of chemokines and cytokines and by promoting differentiation of regulatory T cells [15,16,17]. Its efficacy and safety were not limited to animal studies, exploratory human clinical study involving 90 asymptomatic subjects with atopy (serum total Immunoglobulin E (IgE) > 300 IU/mL) had also shown its potential as a safe immunosuppressive agent [18].

In this study, we investigated the effects of PG102 on murine imiquimod (IMQ)-induced psoriasis-like skin inflammation. Our data showed that PG102 could effectively suppress symptoms of psoriasis-like skin inflammation by inhibiting neutrophil infiltration through the regulation of chemokines, antimicrobial peptides, and NF-κB, signal transducer of activation (STAT) signaling in keratinocytes. These results suggest that PG102 may be a potent and safe agent in the alleviation of psoriasis.

## 2. Materials and Methods

### 2.1. Preparation of PG102

Hardy kiwifruit *Actinidia arguta* was purchased from a company specializing in this fruit (Hurst’s Berry Farm, McMinnville, OR, USA) and was identified by Plant DNA Bank in Korea (Seoul, Korea). PG102 was prepared as previously described [15,16]. Briefly, the dried fruit was extracted in boiling distilled water for 3 h, followed by filtration (No. 2; 110 mm, Whatman International Ltd., Kent, UK). The filtered extract was concentrated by rotary evaporator and lyophilized. This extract was designated as PG102 and its quality was controlled, as previously described [15,16]. Further quality control was employed by measuring the ability of PG102 to suppress IL-8 production in cytokine-stimulated HaCaT cells and half maximal inhibitory concentration (IC_50_) value was compared to that of the reference batch. PG102 (batch #2) stocks were prepared at a concentration of 200 mg/mL in distilled water (DW), stored at −80 °C and used throughout this study.

### 2.2. Cell Culture and Reagents

Human keratinocyte cell line HaCaT was purchased from CLS Cell Lines Service GmbH (Eppelheim, Germany) and cultured in Dulbecco’s modified Eagle’s medium (Thermo Fisher, Waltham, MA, USA) supplemented with 10% fetal bovine serum (FBS, Corning, Corning, NY, USA) and antibiotics (100 U/mL penicillin and 100 mg/mL streptomycin) at 37 °C in a 5% CO_2_ humidified incubator. Human promyelocytic cell line HL-60 was purchased from American Type Culture Collection (ATCC, Manassas, VA, USA) and cultured in Iscove’s Modified Dulbecco’s Medium (IMDM; ATCC) supplemented with 20% FBS and antibiotics (1% penicillin/streptomycin) at 37 °C in a 5% CO_2_ humidified incubator. M5 cytokine mix was made by combining recombinant human tumor necrosis factor (TNF)-α, Oncostatin M, Interleukin (IL)-1α, IL-17A and IL-22, purchased from BioLegend (San Diego, CA, USA). Cells at passage 3-to-5 were used throughout the experiment.

### 2.3. Mice and Treatment

Seven-week-old female BALB/c mice were obtained from Samtako Bio Korea (Osan, Korea) and housed at 23 ± 2 °C with a 12-h light/dark cycle and free access to standard chow and water. Three days prior to treatment, back skin of mice were shaved using electronic shaver and Niclean shaving cream (Ildong Pharmaceuticals, Seoul, Korea). Mice were topically administered with 62.5 mg of 5% Imiquimod (IMQ) cream (Aldara; 3M Health Care Limited, Loughborough, UK) along with 50 µL of dimethyl sulfoxide (DMSO; Sigma-Aldrich, St. Louis, MO, USA) or PG102 reconstituted in Dimethyl sulfoxide DMSO (100 mg/kg) for 6 consecutive days. The selected dose of PG102 was the optimal dose set based on previous experiment. The control mice were treated with DMSO only. Mice were sacrificed and skin tissue samples, lymph nodes, and blood were collected for further analysis. All experimental procedures were performed in compliance with the guidelines set by the Seoul National University Institutional Animal Care and Use Committee (Approval Number: SNU-150923-1-2).

### 2.4. Scoring Severity of Skin Inflammation

Erythema and scaling were scored blindly based on the clinical Psoriasis Area and Severity Index (PASI). Photos of back skin taken each day were distributed randomly to three people for scoring erythema and scaling on a scale from 0 to 4. 0, none; 1, slight; 2, moderate; 3, marked; 4, very marked; and the scores were averaged. The level of erythema was scored using a scoring table with red taints. Back skin thickness was measured every day, from day 0 before treatment, and on day 6, before sacrificing mice using a micrometer (Mitutoyo, Japan). The cumulative score (erythema plus scaling plus thickening) served as a measure of the severity of inflammation (scale 0–12).

### 2.5. Cell Isolation and Stimulation

Axillary and inguinal lymph nodes were aseptically removed from each mouse. Lymph nodes in each experimental groups were pooled and single cell suspensions from lymph nodes were obtained as previously described [19]. Isolated cells were cultured at 1 × 10^6^ cells/well with 50 ng/mL phorbol 12-myristate 13-acetate (PMA) and 500 ng/mL ionomycin (Sigma-Aldrich, Saint Louis, MO, USA) and after 24 h, culture supernatants were collected for further analysis.

### 2.6. Tissue Preparation and Immunohistochemistry (IHC)

Back skin tissue of each mouse was fixed with 10% neutral buffered formalin (Sigma-Aldrich) for at least 24 h and staining was performed in Korean Pathology Technical Center (KP&T, Cheong-ju, Korea). Briefly, samples were embedded in paraffin and sectioned into 3 µm-thick sections, followed by staining with hematoxylin-eosin (H&E). For immunohistochemistry (IHC), sections were incubated with anti-mouse Ly6G antibody (1:1000; Abcam, Cambridge, UK) followed by incubation with biotinylated secondary antibody and developed with 3,3′-diaminobenzidine (DAB). Samples were analyzed with a microscope (Olympus, Tokyo, Japan) and random fields were acquired for each slide. Five random high power field (HPF) images (400×) of each slide were taken and Ly6G positive cells were counted using ImageJ software version 1.50i (National Institutes of Health, Bethesda, MD, USA).

### 2.7. Measurement of Cell Proliferation

Cells were seeded at a density of 5 × 10^4^ cells/well in 24-well cell culture plates overnight (*n* = 3). Cells were then incubated with M5 and PG102 at designated concentrations for 24 and 48 h. After incubation, cell proliferation was assessed by CellVia WST-1 assay according to the manufacturer’s protocol (Young In Frontier, Seoul, Korea).

### 2.8. RNA Extraction and Quantitative Real-Time Polymerase Chain Reaction (RT-PCR)

Cells were seeded at a density of 2 × 10^5^ cells/well in 12-well cell culture plates overnight (*n* = 3) and then treated with M5 and PG102 at designated concentrations for 6 and 24 h. Back skin tissues of mice were cut and kept in RNAlater™ Stabilization Solution (Invitrogen, Waltham, MA, USA) until use. Total RNA was isolated from cells or skin tissues using RNAiso (Takara Bio, Shiga, Japan) according to the manufacturer’s protocol and complementary DNA (cDNA) was synthesized using AMV reverse transcriptase (Takara Bio) and oligo dT primers (Qiagen, Hilden, Germany). Quantitative RT-PCR of each cDNA was performed using SYBR Premix Ex Taq^TM^ (Takara Bio) and Thermal Cycler Dice Real Time System (Takara Bio) with the following protocol: 30 s at 95 °C, followed by 40 cycles of 5 s at 95 °C and 30 s at 60 °C. RNA levels were normalized by the level of HPRT and the relative changes in gene expression were calculated as 2−^ΔΔCt^ method. Primer sequences used in this study are listed in Table 1. Single amplicons were verified for each set of primers.

### 2.9. Enzyme-Linked Immunosorbent Assay (ELISA)

Mouse IL-17A, human S100A8/A9 heterodimer, CXCL1, CXCL5 and interleukin (IL)-8 (R and D Systems, Minneapolis, MN, USA) and β-defensin 2 (Peprotech, Rocky Hill, NJ, USA) in cell culture supernatants were measured by using commercially available enzyme-linked immunosorbent assay (ELISA) kits according to the manufacturer’s instructions.

### 2.10. Western Blot Analysis

Cells were seeded at a density of 1 × 10^6^ cells/plate on 60 mm cell culture dish overnight. Cells were incubated with M5 or PG102 at designated concentrations for 30 min and total cell lysates were extracted with CytoBuster™ (Merck, Darmstadt, Germany) mixed with PhosSTOP™ and cOmplete™ Protease Inhibitor Cocktail (Roche, Basel, Switzerland). Total protein contents in the cell lysates were determined by bicinchoninic acid (BCA) assay kit and after reconstituting in sample buffer, 10 micrograms of protein samples were subjected to SDS-PAGE on Bolt™ 10% Bis-Tris Plus Gels (Invitrogen). Proteins were transferred onto a PVDF membrane (Merck) and the membrane was incubated in 5% skim milk in 0.1% TBST at room temperature for 1 h to block nonspecific binding. The membrane was then incubated with antibodies specific for phospho-STAT3 (#9134, #9145), STAT1 (#9167), p65 (#3033), and STAT3 (#4904), STAT1 (#9172), p65 (#8242), IκB-α (#9242) (1:1000; Cell Signaling Technology, Danvers, MA, USA), and β-actin (A5441, Sigma-Aldrich) overnight at 4 °C followed by incubation with horseradish peroxidase-conjugated secondary anti-mouse or anti-rabbit IgG (1:100,000; Sigma-Aldrich) at room temperature for 1 h. The blot was developed by Immobilon ECL HRP substrate (Merck) and visualized by exposure on autoradiography film.

### 2.11. Neutrophil Chemotaxis Assay

To differentiate HL-60, cells were cultured in medium containing 1.3% DMSO for 5 days, as previously reported [20]. Differentiated HL-60 (dHL-60) cells were washed twice with serum free medium and chemotaxis assays were performed using 3 µm CytoSelect™ Cell Migration Assay Kit (Cell Biolabs, San Diego, CA, USA), according to the manufacturer’s instructions. Briefly, 2 × 10^5^ cells/well were seeded onto the upper membrane chamber and cell culture supernatants from HaCaT cells incubated with M5 and various concentrations of PG102 were added to the bottom wells. After 2 h, migrated cells in the bottom wells were lysed and fluorescence was read at 480 nm/520 nm using FlexStation 3 microplate reader (Molecular Devices, San Jose, CA, USA).

### 2.12. Statistical Analysis

The data are presented as the mean ± standard deviation (SD) of triplicate measurements for in vitro experiments and mean ± standard error of the mean (SEM) for in vivo experiments. Each experiment was repeated at least three times, independently. Data analysis was performed using the GraphPad Prism version 6.0 (GraphPad Software, San Diego, CA, USA). Comparisons with other experimental groups were analyzed by either one-way analysis of variance (ANOVA) followed by the Bonferroni post-hoc test. *p*-Values less than 0.05 were considered statistically significant.

## 3. Results

### 3.1. Quality of PG102 is Standardized by Quantification of Marker Compounds and Cell-Based IL-8 Bioassay

**Figure 1:**
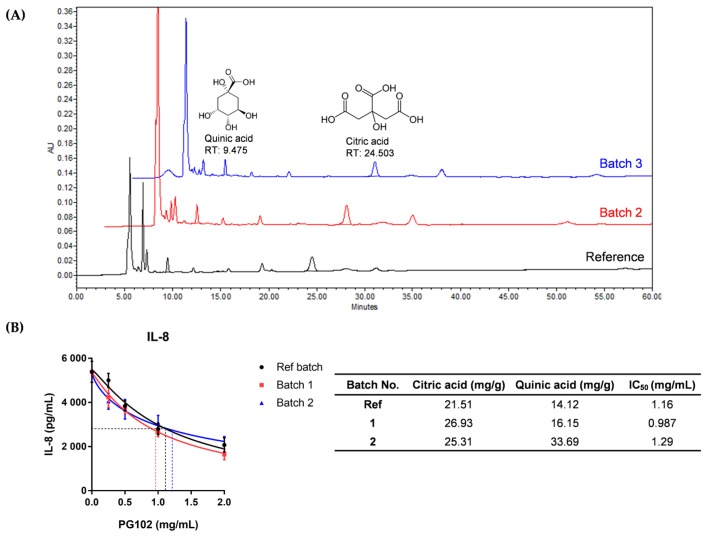
PG102 is a standardized extract from Actinidia arguta. (**A**) Quantification of marker compounds citric acid and quinic acid; (**B**) Interleukin (IL)-8 bioassay and half maximal inhibitory concentration (IC_50_) of PG102 in HaCaT cells. Ref: reference. RT: retention time.

To establish batch-to-batch consistency of extracts prepared from *A. arguta*, its quality has been standardized as described previously [15]. First, the contents of two marker compounds citric acid and quinic acid were quantified using high performance liquid chromatography (HPLC). Only the extracts containing these compounds within standard range (19.0–29.0 mg/g for citric acid; 14.0–22.0 mg/g for quinic acid) were chosen. As shown in Figure 1, two different batches of PG102 were compared with the reference batch. Since batch 3 contained citric acid and quinic acid out of standard range, it has been discarded.

In addition, a cell-based bioassay was employed using IL-8, which is a relevant biomarker of psoriasis [21]. Keratinocyte cell line HaCaT cells were stimulated with a cytokine mixture called M5 (consisting of IL-1α, IL-17A, IL-22, TNF-α and Oncostatin M) to mimic conditions of psoriatic keratinocytes [22]. When HaCaT cells were treated with M5 and PG102, IL-8 level in the supernatant was decreased in a dose-dependent manner by PG102. The half maximal inhibitory concentration IC_50_ of batch 2 was 0.987 mg/mL, similar to that of the reference batch (1.16 mg/mL) (Figure 1B). While the IC_50_ value of batch 3 was not significantly different from that of the reference batch, it was not used as its contents of marker compounds did not fall within the standard range. All batches of PG102 did not have any cytotoxic effects in any of the concentrations used in these experiments (Figure S1). The contents of marker compounds citric acid and quinic acid of PG102 and IC_50_ values of many different batches of PG102 had been remarkably similar. The use of two quality control assays ensured not only its batch-to-batch chemical composition, but also its biological activities.

### 3.2. Topical Application of PG102 Ameliorates Clinical Symptoms of IMQ-Induced Psoriasis-Like Skin Inflammation

**Figure 2:**
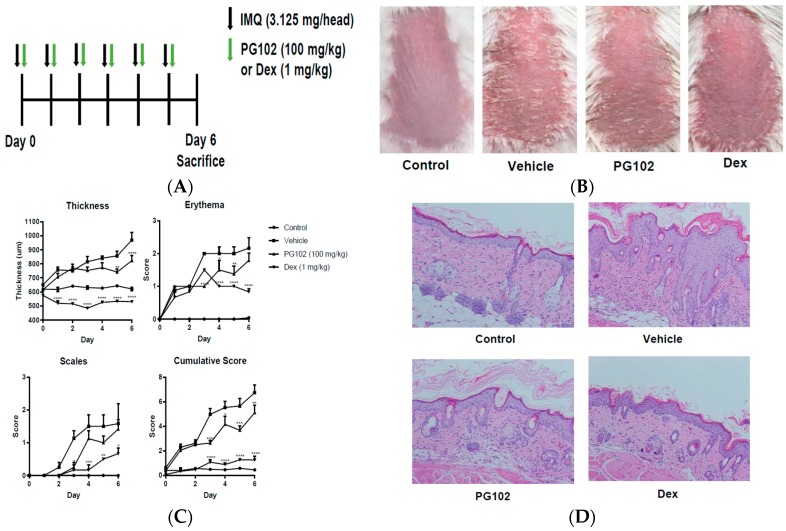

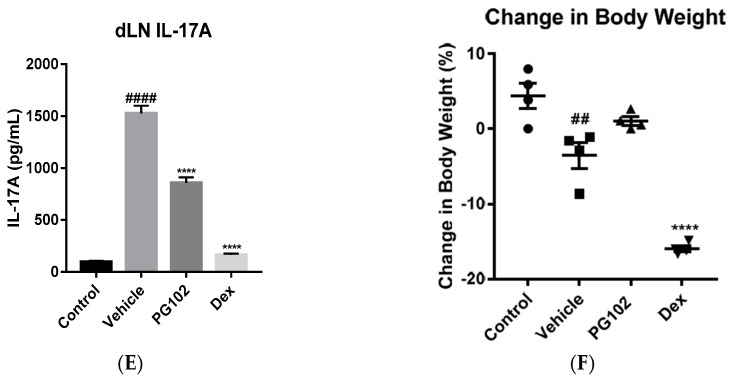
Topical treatment of PG102 alleviates imiquimod (IMQ)-induced psoriasis-like symptoms. (**A**) Experimental scheme (**B**) Photo of dorsal skin of Control (DMSO only), Vehicle (IMQ + DMSO), PG102 (IMQ + PG102 100 mg/kg) and Dexamethasone (IMQ + Dex 1 mg/kg) treated mice. (**C**) Psoriasis Area and Severity Index (PASI) scoring. (**D**) Hematoxylin-eosin (H&E) staining of dorsal skin (200×). (**E**) Interleukin (IL)-17A protein level from phorbol 12-myristate 13-acetate (PMA)/ionomycin-restimulated cells isolated from draining lymph nodes. (**F**) Change in body weight. Representative results from at least three independent experiments are shown (*n* = 3–4). The data are shown as the mean ± standard error of mean (SEM). ^##^ *p* < 0.01, ^####^ *p* < 0.0001 versus Control group; * *p* < 0.05, ** *p* < 0.01, *** *p* < 0.001, **** *p* < 0.0001 versus vehicle group.

The effects of PG102 were investigated in the IMQ-induced psoriasis-like skin inflammation model. Aldara cream, containing TLR7 agonist IMQ, has been used to induce psoriasis-like inflammation characterized by increase in epidermal thickness, erythema and scaling [23]. Topical application of PG102 for six consecutive days improved clinical scores of psoriasis, mainly that of skin thickness (Figure 2B,C). H and E staining of the back skin clearly showed reduced epidermal thickness compared to the vehicle group (Figure 2D). The levels of IL-17A from cells isolated from draining lymph nodes (dLN) and serum were decreased in PG102-treated mice (Figure 2E and Figure S2). Treatment with the positive control dexamethasone showed significant suppression of inflammatory responses, but exerted severe adverse effects such as skin atrophy and weight loss (Figure 2C,F). These results suggest that topical treatment with PG102 could alleviate both local and systemic inflammation in IMQ-induced psoriasis-like inflammation in mice without causing adverse effects.

### 3.3. PG102 Inhibits Hyperproliferation of Hacat Cells and Phosphorylation of STAT3

**Figure 3:**
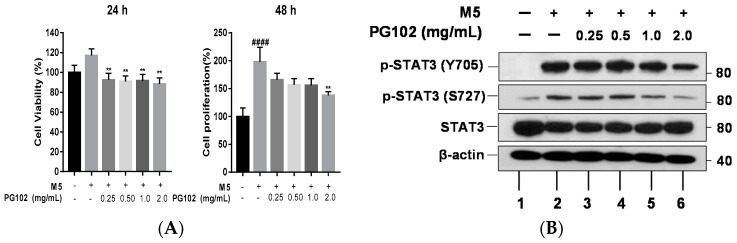

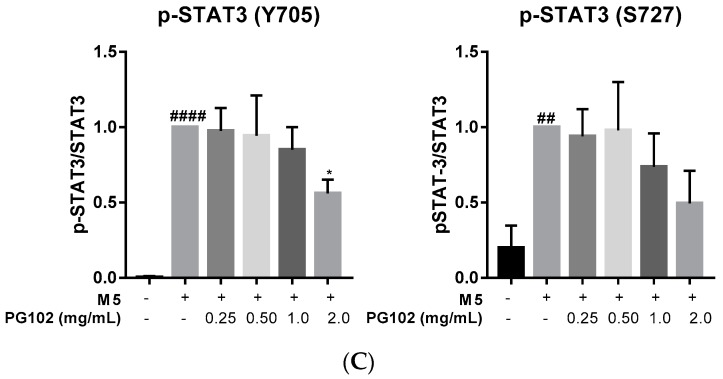
PG102 suppresses hyperproliferation of keratinocytes through STAT3 signaling. (**A**) WST-1 cell proliferation assay in HaCaT cells treated with M5 and PG102 for 24 h and 48 h (*n* = 3). (**B**) PG102 was treated to M5-stimulated HaCaT cells for 30 min and Western blot analysis was performed. (**C**) Densitometry results of Western blot. The means of three independent experiments are shown. p-STAT3 (Y705), phospho-signal transducer and activator of transcription 3 (Tyr705). p-STAT3 (S727), phospho-signal transducer and activator of transcription 3 (phospho S727). ^##^ *p* < 0.01, ^####^ *p* < 0.0001 versus Control group; * *p* < 0.05, ** *p* < 0.01 versus M5-only treated group. The data are shown as the mean ± standard error (SD).

Since hyperproliferation of keratinocytes is one of the main manifestations of psoriatic lesions, anti-proliferative effects of PG102 were assessed. To evaluate effects of PG102 on proliferation of keratinocytes, HaCaT cells were treated with M5 and PG102 for 24 and 48 h. The M5-stimulated group showed significant increase in proliferation at 48 h, while treatment with PG102 suppressed the proliferation (Figure 3A).

Next, we corroborated the effects of PG102 on STAT3 signaling, whose activation has been known to induce proliferation of keratinocytes [24]. Consistent with previous reports, M5 treatment increased phosphorylation of STAT3 at both tyrosine 705 and serine 727 residues. Treatment with PG102 at 2.0 mg/mL effectively reduced their expression levels, although the degree of suppression was not statistically significant for phosphorylation of serine 727 residue (Figure 3B,C). These results indicated that the effect on epidermal thickness is, in part, due to the suppression of STAT3 phosphorylation and hyperproliferation of keratinocytes (Figure 2C).

### 3.4. PG102 Downregulates Expression of Chemokines in HaCaT Cells

**Figure 4:**
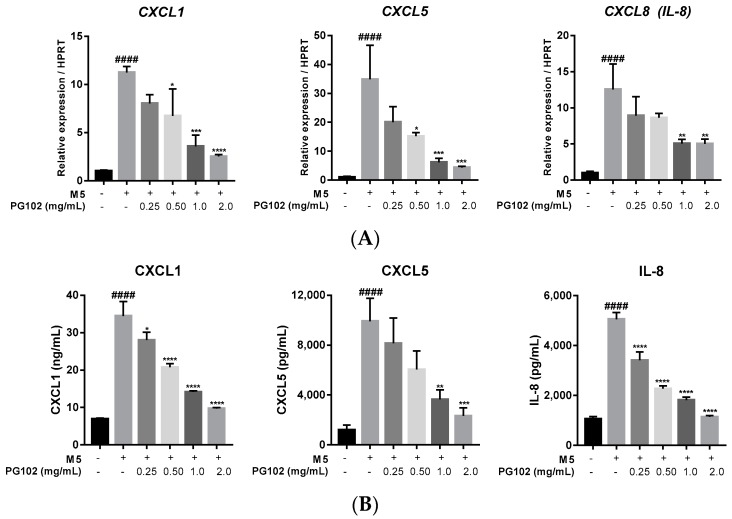
PG102 downregulates expression of chemokines on M5-stimulated HaCaT cells in a dose-dependent manner. (**A**) Real-time polymerase chain reaction (PCR) (qPCR) analysis of mRNA expression levels of chemokines after 6 h. (**B**) Protein levels of chemokines measured in the cultured supernatant by enzyme-linked immunosorbent assay (ELISA) after 24 h. CXCL, chemokine (C-X-C motif) ligand. ^####^ *p* < 0.001 versus negative control group; * *p* < 0.05, ** *p* < 0.01, *** *p* < 0.001, **** *p* < 0.0001 versus M5-only treated group.

In psoriatic epidermis, keratinocytes actively participate in the immune responses by releasing pro-inflammatory cytokines, chemokines, and antimicrobial peptides to recruit various immune cells [1]. Thus, blocking expression of these inflammatory mediators has beneficial roles in regulating psoriasis. CXCL1, CXCL5, and IL-8 are chemokines released by keratinocytes and they recruit neutrophils through interactions with CXCR2 and CXCR1, respectively [25]. In M5-stimulated HaCaT cells, their mRNA and protein levels are dramatically increased but treatment with PG102 effectively downregulated expressions of these chemokines in a concentration-dependent manner (Figure 4A,B). These data showed that PG102 might exert its biological activities through the negative regulation of chemokines.

### 3.5. PG102 Significantly Suppresses Expression of Antimicrobial Peptides in HaCaT Cells

**Figure 5:**
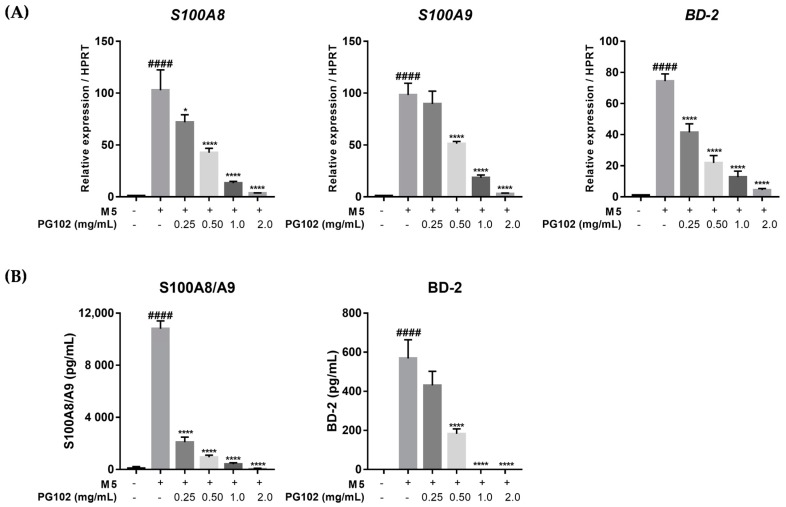
PG102 downregulates expression of antimicrobial peptides on M5-stimulated HaCaT cells in a dose-dependent manner. (**A**) Real-time PCR (qPCR) analysis of mRNA expression levels of antimicrobial peptides after 24 h. (**B**) Protein levels of antimicrobial peptides measured in the cultured supernatant by ELISA after 48 h. BD-2, beta-defensin 2; S100, S100 calcium binding protein. ^####^ *p* < 0.0001 versus negative control group; * *p* < 0.05, **** *p* < 0.0001 versus M5-only treated group.

Next, it was assessed whether PG102 could suppress the expression of antimicrobial peptides in keratinocytes. S100A8 and S100A9 are antimicrobial peptides that form a heterodimer called calprotectin which interacts with Toll-like receptor 4 (TLR-4) and exacerbates psoriasis by activating complement factor C3 and recruiting neutrophils [9,26]. β-Defensin 2, a serum biomarker for psoriasis, is another antimicrobial peptide that is known to induce chemotaxis of immune cells through interaction with CC chemokine receptor 2 (CCR2) [8]. In the M5-treated group, the mRNA levels of S100A8, S100A9, and β-defensin 2 (hBD-2) were highly increased, but treatment with PG102 reduced their levels in a dose-dependent manner without cytotoxic effects (Figure 5A). Similar observations were made when protein levels of S100A8/A9 heterodimer and hBD-2 were measured in the supernatant, suggesting that PG102 might regulate the expression of antimicrobial peptides at both mRNA and protein levels (Figure 5B).

### 3.6. PG102 Exerts Anti-Inflammatory Effects through Inhibition of STAT and NF-κB Signaling

**Figure 6:**
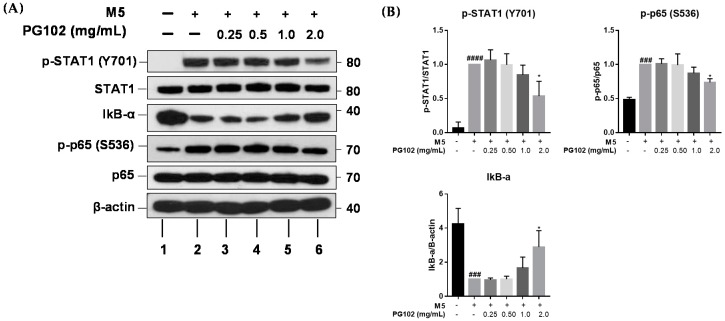
PG102 inhibits NF-κB and STAT1 signaling in HaCaT cells. (**A**) Western blot results of corresponding proteins after treatment with M5 and PG102 for 30 min. (**B**) Densitometry of Western blot. The means of three experiments are shown. ^###^ *p* < 0.001, ^####^ *p* < 0.0001 versus Control group; * *p* < 0.05 versus M5-only treated group.

Transcription of chemokines and antimicrobial peptides involved in psoriasis has been reported to depend largely on STAT and NF-κB signalings [27,28]. In an effort to understand molecular mechanisms underlying the anti-inflammatory activities of PG102, HaCaT cells were co-treated with M5 and PG102 for 30 min, and the levels of various signaling proteins were measured by Western blot. M5 stimulation increased the level of phosphorylated STAT1, while co-treatment with PG102 lowered its amount in a concentration-dependent manner. M5 also induced degradation of IκB-α and subsequent phosphorylation of p65, while PG102 treatment at 2.0 mg/mL significantly prevented degradation of IκB-α and phosphorylation of p65 (Figure 6). These results demonstrate that PG102 exerts anti-inflammatory activities through inhibition of phosphorylation of STAT1, NF-κB p65, and degradation of IκB-α.

### 3.7. PG102 Suppresses Neutrophil Chemotaxis in vitro and Neutrophil Infiltration to Skin in IMQ-Treated Mice

**Figure 7:**
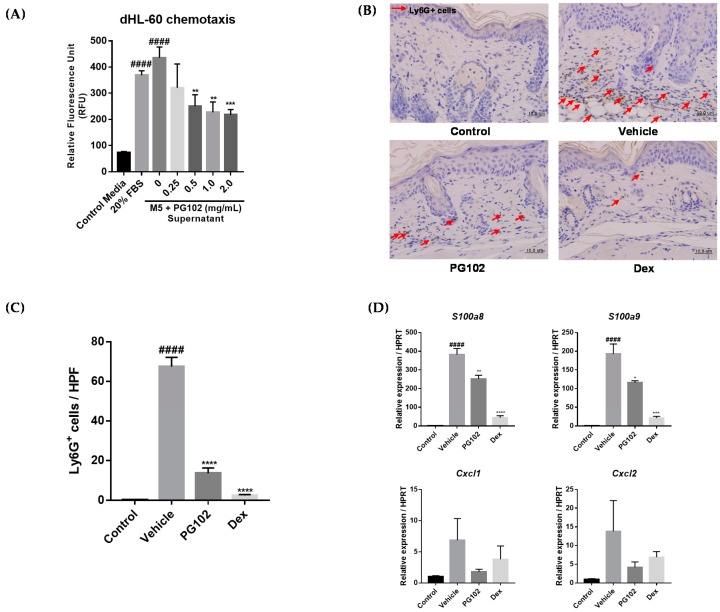
PG102 suppresses neutrophil chemotaxis in vitro and in vivo. (**A**) Chemotaxis assay of differentiated HL-60 (dHL-60) cells. (**B**) IHC staining for Ly6G in dorsal skin of control, vehicle, PG102 (100 mg/kg), Dex (1 mg/kg) treated mice. (**C**) Quantification of Ly6G+ cells per five random high power fields (400X). (**D**) Real-time qPCR analysis of mRNA expression levels of antimicrobial peptides and chemokines in dorsal skin of mice (*n* = 3–4). FBS, fetal bovine serum; Dex, dexamethasone; M5, mixture of 5 cytokines. The data are shown as the mean ± SEM. ^####^ *p* < 0.0001 versus Control group; * *p* < 0.05, ** *p* < 0.01, *** *p* < 0.001, **** *p* < 0.0001 versus vehicle group.

One of the hallmarks of psoriatic lesions is the marked infiltration of neutrophils, among many other immune cells [2]. We hypothesized that PG102 might exert its anti-psoriatic effects by regulating the expression of chemokines and antimicrobial peptides and inhibiting subsequent neutrophil infiltration. To test this hypothesis, HL-60, a neutrophil-like cell line, was subjected to chemotaxis assay. Differentiated HL-60 cells (dHL-60) were placed in the upper well of the Boyden chamber, in the presence of cell culture supernatants from HaCaT cells treated with M5 and PG102 for 48 h in the bottom well. Medium containing 20% FBS served as the positive control. The number of HL-60 cells migrated to the bottom well were measured by fluorescence and PG102-treated supernatant resulted in a lower number of migrated HL-60 cells in a concentration-dependent manner (Figure 7A).

The extent of neutrophil infiltration to dorsal skin in IMQ-treated mice was measured by immunostaining of the lymphocyte antigen 6 complex locus G (Ly6G) protein. In the vehicle-treated group, the number of Ly6G^+^ cells were greatly increased in the dermal layer of the skin. However, treatment with PG102 or dexamethasone suppressed neutrophil infiltration to the skin, along with reduced thickness of the epidermis (Figure 7B,C). The RNA levels of CXCL1, CXCL2, S100A8, and S100A9 were also reduced in both PG102 and dexamethasone-treated groups (Figure 7D). Taken together, these data suggested that PG102 might ameliorate IMQ-induced psoriasis-like skin inflammation by suppressing neutrophil chemotaxis induced by inflamed keratinocytes.

## 4. Discussion

PG102 is a botanical extract derived from an edible portion of *Actidinia arguta*. It has previously been shown to contain potent anti-inflammatory and anti-oxidative activities both in vitro and in vivo [15,16,17,29,30]. Based on these reports, we investigated the effect of PG102 on the murine IMQ-induced psoriasis-like skin inflammation model. As PG102 consists a mixture of compounds, we initially established two quality control methods to minimize batch-to-batch variations. First, the contents of citric acid and quinic acid were quantified by HPLC; as these compounds are not found exclusively in *Actinidia arguta*, two marker compounds were used for quality control. Next, each batch of PG102 was subjected to IL-4 bioassay to ensure its bioactivity. Here, an additional bioassay using IL-8 ELISA was employed as this cytokine is a more relevant cytokine in psoriasis. Only batches which satisfied the standards for both assays were used. The quality of PG102 was found to be remarkably consistent in different batches. In this study, we demonstrated that topical application of PG102 ameliorates clinical symptoms of psoriasis by inhibition of STAT3-mediated proliferation of keratinocytes and neutrophil infiltration to skin.

One of the hallmark features of psoriasis is epidermal thickening caused by hyperproliferation of keratinocytes along with abnormal differentiation of keratinocytes, which ultimately leads to dysfunctional skin barrier [31]. IL-22, produced by Th17 and Th22 cells, is the key cytokine responsible for hyperproliferation of keratinocytes and disruption of terminal differentiation by activating STAT3 [24]. Thus, STAT3 has been aroused as an ideal therapeutic target for psoriasis. Indeed, a recent study has shown that calcipotriol, which has been used widely for the treatment of psoriasis, inhibits proliferation of keratinocytes by downregulating phosphorylation of STAT3 [32]. Our data showed a decrease in epidermal thickness in IMQ-treated mice by topical application of PG102 as well as suppression of proliferation and phosphorylation of tyrosine 705 and serine 727 residues of STAT3 in HaCaT cells. Besides, we have observed an increase in late differentiation markers, such as filaggrin and involucrin, by treatment with PG102 in HaCaT cells, suggesting possible therapeutic roles of PG102 on differentiation of keratinocytes.

Another hallmark of psoriasis is the marked infiltration of neutrophils into epidermis that form microabscesses and neutrophil extracellular traps (NETs) composed of DNA and AMPs [33]. While many of the drugs developed for psoriasis primarily focus on the IL-23/Th17 axis of the disease, the roles of neutrophils should not be overlooked, as neutrophils are one of the most predominant cell types in psoriatic skin [34]. Indeed, it has been shown that depletion of neutrophils rapidly improved the symptoms of psoriasis while recovery of neutrophils reversed this effect [35]. Pathological significance of neutrophils has been confirmed by the observation that RAR-related orphan receptor gamma t (RORγt), the key transcriptional regulator of IL-17A, is expressed in neutrophils present in psoriatic lesions [36]. Moreover, neutrophils were identified as the numerically largest source of IL-17A in psoriasis along with Th17 cells [34]. Thus, targeting neutrophil trafficking appears to be a viable approach to relieve psoriasis. Our results clearly showed that topical application of PG102 can reduce both neutrophil infiltration to skin and IL-17A production from the cells present in the draining lymph node in this study, we have not identified the IL-17A-producing cells affected by PG102. Reduction in IL-17A level could be due to direct suppression of IL-17A-producing cells or due to upregulation of anti-inflammatory mediators. The latter may be a possible explanation based on the previous report describing an increase in regulatory T cell generation by PG102 [17]. It would be worth further investigating the cellular target of PG102 in psoriasis-like skin inflammation.

In the current study, we also observed dramatic downregulation of neutrophil-chemotactic chemokines and antimicrobial peptides at both RNA and protein levels by treatment with PG102. It is well documented that the canonical NF-κB signaling, which initiates by degradation of IκB-α and subsequent phosphorylation and translocation of NF-κB p65 subunit, is involved in the transcription of numerous chemokines, including CXCL1, CXCL5, and IL-8 [37]. Moreover, promoters of S100A8 and S100A9 include binding sites for NF-κB and STAT3, while that of β-defensin 2 contains binding sites for NF-κB and STAT1 [28,38]. To understand the molecular mechanism of PG102, we analyzed the effects of PG102 on STAT1 and NF-κB p65 phosphorylation in M5-stimulated HaCaT cells. Our data suggested that treatment with PG102 effectively inhibited phosphorylation of these signaling molecules, in accordance to downregulation of chemokines and antimicrobial peptides.

Identification of active compounds and additional cellular targets of PG102 remain to be elucidated. The effects of PG102 on NF-κB, STAT signaling pathway could be due to simultaneous actions of multiple components of PG102 or selective targeting of upstream regulators. Given the high degrees of safety and efficacy of PG102 in the psoriasis-like model, further investigation is warranted to identify and isolate active compounds responsible for its biological activities to facilitate its quality control measures.

## 5. Conclusions

Topical application of PG102 alleviated IMQ-induced psoriasis-like skin inflammation by suppressing proliferation of keratinocytes and negatively regulating chemotaxis of neutrophils, which are induced by inflammatory mediators released from keratinocytes. Given its distinctive biological activities, PG102 may provide a safe and effective means of treating psoriasis.

## Figures and Tables

**Table 1:** Sequences of primers used for quantitative real-time polymerase chain reaction (PCR).

Target Gene	Primer Sequence (5′-3′)	
	Forward	Reverse
*hBD-2*	5′-GGTGTTTTTGGTGGTATAGGC-3′	5′-AGGGCAAAAGACTGGATGACA-3′
*hCXCL1*	5′-AATCCTGCATCCCCCATA-3′	5′-TGTCTCTCTTTCCTCTTCTGTTCCT-3′
*hCXCL5*	5′-TGCGTTGCGTTTGTTTACAG-3′	5′-GAAAAGGGGCTTCTGGATCA-3′
*hCXCL8 (IL-8)*	5′-ATGACTTCCAAGCTGGCCGTG-3′	5′-TTATGAATTCTCAGCCCTCTTCAAAAACTTCTC-3′
*hHPRT*	5′-TATGGCGACCCGCAGCCCT-3′	5′-CATCTCGAGCAAGACGTTCAG-3′
*hS100A8*	5′-GGGAATTTCCATGCCGTCT-3′	5′-CCTTTTTCCTGATATACTGAGGAC-3′
*hS100A9*	5′-CAGCTGGAACGCAACATAGA-3′	5′-TCAGCTGCTTGTCTGCATTT-3′
*mCXCL1*	5′-CACCTCAAGAACATCCAGAGCT-3′	5′-CAAGCAGAACTGAACTACCATCG-3′
*mDEFB4*	5′-GCTTCAGTCATGAGGATCCAT-3′	5′-CTTGCTGGTTCTTCGTCTTTT-3′
*mHPRT*	5′-CACAGGACTAGAACACCTGC-3′	5′-GCTGGTGAAAAGGACCTCT-3′
*mCXCL2*	5′-TTGCCTTGACCCTGAAGCCCCC-3′	5′-GGCACATCAGGTACGATCCAGGC-3′
*mS100A8*	5′-AAATCACCATGCCCTCTACAAG-3′	5′-CCCACTTTTATCACCATCGCAA-3′
*mS100A9*	5′-ATACTCTAGGAAGGAAGGACACC-3′	5′-TCCATGATGTCATTTATGAGGGC-3′

BD-2, beta-defensin 2; CXCL: C-X-C motif ligand; IL, interleukin, HPRT, hypoxanthine-guanine phosphoribosyltransferase; S100, S100 calcium binding protein; DEFB4, defensin beta 4; h, human; m, mouse; F, forward, R, reverse.

## Supplementary Materials

The following are available online at http://www.mdpi.com/2072-6643/10/10/1399/s1, Figure S1: Effects of PG102 on cell viability of HaCaT cells. HaCaT cells were treated with different concentrations of PG102 for 24 h and were subjected to cell viability assay. The data are shown as the mean ± SD, Figure S2: Topical administration of PG102 reduces IL-17A level in serum. After 6 days of IMQ treatment, serum from mice were collected were subjected to ELISA (*n* = 6). The data are shown as the mean ± SEM. ^####^ *p* < 0.0001 versus Control group; **** *p* < 0.0001 versus vehicle group.

## References

[1] Greb J.E., Goldminz A.M., Elder J.T., Lebwohl M.G., Gladman D.D., Wu J.J., Mehta N.N., Finlay A.Y., Gottlieb A.B. (2016). Psoriasis. Nat. Rev. Dis. Primers.

[2] Boehncke W.H., Schon M.P. (2015). Psoriasis. Lancet.

[3] Gottlieb A.B., Chao C., Dann F. (2008). Psoriasis comorbidities. J. Dermatol. Treat..

[4] De Korte J., Sprangers M.A., Mombers F.M., Bos J.D. (2004). Quality of life in patients with psoriasis: A systematic literature review. J. Investig. Dermatol. Symp. Proc..

[5] Griffiths C.E.M., van der Walt J.M., Ashcroft D.M., Flohr C., Naldi L., Nijsten T., Augustin M. (2017). The global state of psoriasis disease epidemiology: A workshop report. Br. J. Dermatol..

[6] Lande R., Gregorio J., Facchinetti V., Chatterjee B., Wang Y.H., Homey B., Cao W., Wang Y.H., Su B., Nestle F.O. (2007). Plasmacytoid dendritic cells sense self-DNA coupled with antimicrobial peptide. Nature.

[7] Fitch E., Harper E., Skorcheva I., Kurtz S.E., Blauvelt A. (2007). Pathophysiology of psoriasis: Recent advances on IL-23 and Th17 cytokines. Curr. Rheumatol. Rep..

[8] Rohrl J., Yang D., Oppenheim J.J., Hehlgans T. (2010). Human beta-defensin 2 and 3 and their mouse orthologs induce chemotaxis through interaction with CCR2. J. Immunol..

[9] Lee Y., Jang S., Min J.K., Lee K., Sohn K.C., Lim J.S., Im M., Lee H.E., Seo Y.J., Kim C.D. (2012). S100a8 and s100a9 are messengers in the crosstalk between epidermis and dermis modulating a psoriatic milieu in human skin. Biochem. Biophys. Res. Commun..

[10] Hu S.C.-S., Yu H.S., Yen F.L., Lin C.L., Chen G.-S., Lan C.-C.E. (2016). Neutrophil extracellular trap formation is increased in psoriasis and induces human beta-defensin-2 production in epidermal keratinocytes. Sci. Rep..

[11] Uva L., Miguel D., Pinheiro C., Antunes J., Cruz D., Ferreira J., Filipe P. (2012). Mechanisms of action of topical corticosteroids in psoriasis. Int. J. Endocrinol..

[12] Carrascosa J.M., van Doorn M.B., Lahfa M., Nestle F.O., Jullien D., Prinz J.C. (2014). Clinical relevance of immunogenicity of biologics in psoriasis: Implications for treatment strategies. J. Eur. Acad. Dermatol. Venereol..

[13] Matich A.J., Young H., Allen J.M., Wang M.Y., Fielder S., McNeilage M.A., MacRae E.A. (2003). Actinidia arguta: Volatile compounds in fruit and flowers. Phytochemistry.

[14] Leontowicz H., Leontowicz M., Latocha P., Jesion I., Park Y.S., Katrich E., Barasch D., Nemirovski A., Gorinstein S. (2016). Bioactivity and nutritional properties of hardy kiwi fruit actinidia arguta in comparison with actinidia deliciosa ‘hayward’ and actinidia eriantha ‘bidan’. Food Chem..

[15] Park E.J., Kim B., Eo H., Park K., Kim Y., Lee H.J., Son M., Chang Y.S., Cho S.H., Kim S. (2005). Control of igE and selective T(H)1 and T(H)2 cytokines by PG102 isolated from actinidia arguta. J. Allergy Clin. Immunol..

[16] Kim D., Kim S.H., Park E.J., Kang C.Y., Cho S.H., Kim S. (2009). Anti-allergic effects of PG102, a water-soluble extract prepared from actinidia arguta, in a murine ovalbumin-induced asthma model. Clin. Exp. Allergy.

[17] Bae M.J., Lim S., Lee D.S., Ko K.R., Lee W., Kim S. (2016). Water soluble extracts from actinidia arguta, PG102, attenuates house dust mite-induced murine atopic dermatitis by inhibiting the mTOR pathway with Treg generation. J. Ethnopharmacol..

[18] Kim S.H., Kim S., Lee S.H., Park H.W., Chang Y.S., Min K.U., Cho S.H. (2011). The effects of PG102, a water-soluble extract from actinidia arguta, on serum total ige levels: A double-blind, randomized, placebo-controlled exploratory clinical study. Eur. J. Nutr..

[19] Link A., Vogt T.K., Favre S., Britschgi M.R., Acha-Orbea H., Hinz B., Cyster J.G., Luther S.A. (2007). Fibroblastic reticular cells in lymph nodes regulate the homeostasis of naive t cells. Nat. Immunol..

[20] Millius A., Weiner O.D. (2009). Chemotaxis in neutrophil-like hl-60 cells. Methods Mol. Biol..

[21] Gillitzer R., Berger R., Mielke V., Muller C., Wolff K., Stingl G. (1991). Upper keratinocytes of psoriatic skin lesions express high levels of NAP-1/IL-8 mrna in situ. J. Investig. Dermatol..

[22] Guilloteau K., Paris I., Pedretti N., Boniface K., Juchaux F., Huguier V., Guillet G., Bernard F.X., Lecron J.C., Morel F. (2010). Skin inflammation induced by the synergistic action of IL-17a, IL-22, oncostatin M, IL-1{alpha}, and TNF-{alpha} recapitulates some features of psoriasis. J. Immunol..

[23] Van der Fits L., Mourits S., Voerman J.S., Kant M., Boon L., Laman J.D., Cornelissen F., Mus A.M., Florencia E., Prens E.P. (2009). Imiquimod-induced psoriasis-like skin inflammation in mice is mediated via the IL-23/IL-17 Axis. J. Immunol..

[24] Orecchia V., Regis G., Tassone B., Valenti C., Avalle L., Saoncella S., Calautti E., Poli V. (2015). Constitutive stat3 activation in epidermal keratinocytes enhances cell clonogenicity and favours spontaneous immortalization by opposing differentiation and senescence checkpoints. Exp. Dermatol..

[25] Ha H., Debnath B., Neamati N. (2017). Role of the CXCL8-CXCR1/2 axis in cancer and inflammatory diseases. Theranostics.

[26] Schonthaler H.B., Guinea-Viniegra J., Wculek S.K., Ruppen I., Ximenez-Embun P., Guio-Carrion A., Navarro R., Hogg N., Ashman K., Wagner E.F. (2013). S100a8–S100a9 protein complex mediates psoriasis by regulating the expression of complement factor C3. Immunity.

[27] Wehkamp J., Harder J., Wehkamp K., Wehkamp-von Meissner B., Schlee M., Enders C., Sonnenborn U., Nuding S., Bengmark S., Fellermann K. (2004). Nf-kappab- and AP-1-mediated induction of human beta defensin-2 in intestinal epithelial cells by escherichia coli nissle 1917: A novel effect of a probiotic bacterium. Infect. Immun..

[28] Hsu K., Chung Y.M., Endoh Y., Geczy C.L. (2014). Tlr9 ligands induce S100a8 in macrophages via a stat3-dependent pathway which requires IL-10 and PGE2. PLoS ONE.

[29] Park E.J., Park K.C., Eo H., Seo J., Son M., Kim K.H., Chang Y.S., Cho S.H., Min K.U., Jin M. (2007). Suppression of spontaneous dermatitis in NC/Nga murine model by PG102 isolated from actinidia arguta. J. Investig. Dermatol..

[30] Kim D., Choi J., Kim M.J., Kim S.H., Cho S.H., Kim S. (2013). Reconstitution of anti-allergic activities of PG102 derived from actinidia arguta by combining synthetic chemical compounds. Exp. Biol. Med..

[31] Wolf R., Orion E., Ruocco E., Ruocco V. (2012). Abnormal epidermal barrier in the pathogenesis of psoriasis. Clin. Dermatol..

[32] Liang W., Lin Z., Zhang L., Qin X., Zhang Y., Sun L. (2017). Calcipotriol inhibits proliferation of human keratinocytes by downregulating stat1 and stat3 signaling. J. Investig. Med..

[33] Delgado-Rizo V., Martinez-Guzman M.A., Iniguez-Gutierrez L., Garcia-Orozco A., Alvarado-Navarro A., Fafutis-Morris M. (2017). Neutrophil extracellular traps and its implications in inflammation: An overview. Front. Immunol..

[34] Reich K., Papp K.A., Matheson R.T., Tu J.H., Bissonnette R., Bourcier M., Gratton D., Kunynetz R.A., Poulin Y., Rosoph L.A. (2015). Evidence that a neutrophil-keratinocyte crosstalk is an early target of IL-17a inhibition in psoriasis. Exp. Dermatol..

[35] Toichi E., Tachibana T., Furukawa F. (2000). Rapid improvement of psoriasis vulgaris during drug-induced agranulocytosis. J. Am. Acad. Dermatol..

[36] Keijsers R.R.M.C., Hendriks A.G.M., van Erp P.E.J., van Cranenbroek B., van de Kerkhof P.C.M., Koenen H.J.P.M., Joosten I. (2014). In vivo induction of cutaneous inflammation results in the accumulation of extracellular trap-forming neutrophils expressing rorgammat and IL-17. J. Investig. Dermatol..

[37] Richmond A. (2002). Nf-kappa B, chemokine gene transcription and tumour growth. Nat. Rev. Immunol..

[38] Albanesi C., Fairchild H.R., Madonna S., Scarponi C., De Pita O., Leung D.Y., Howell M.D. (2007). IL-4 and IL-13 negatively regulate TNF-alpha- and IFN-gamma-induced beta-defensin expression through stat-6, suppressor of cytokine signaling (socs)-1, and socs-3. J. Immunol..

