# Supplementary material for: A Water-Soluble Extract from Actinidia arguta Ameliorates Psoriasis-Like Skin Inflammation in Mice by Inhibition of Neutrophil Infiltration
Source: Nutrients. 2018 Oct 2;10(10):1399. doi: 10.3390/nu10101399 (PMC6213123; doi:10.3390/nu10101399)

Fig. S1. Effects of PG102 on cell viability of HaCaT cells. HaCaT cells were treated with different concentrations of PG102 for 24 hours and were subjected to cell viability assay. The data are shown as the mean ± S.D.

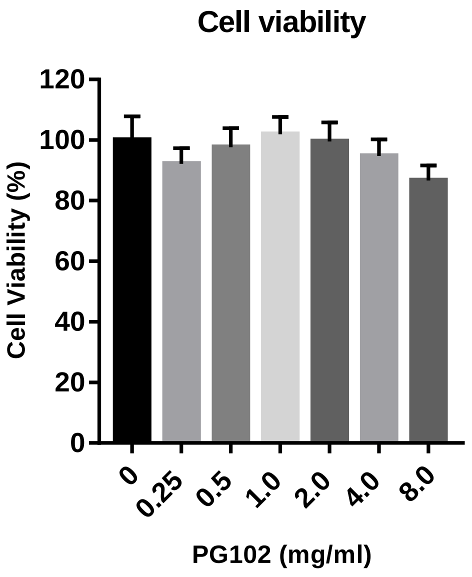

Fig. S2. Topical administration of PG102 reduces IL-17A level in serum. After 6 days of IMQ treatment, serum from mice were collected were subjected to ELISA (n=6). The data are shown as the mean ± S.E.M. #### P<0.0001 versus Control group; **** P<0.0001 versus vehicle group.

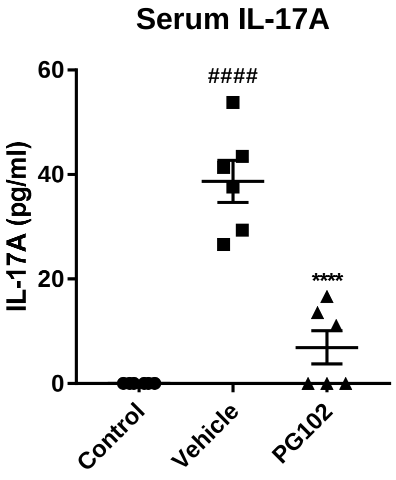

Supplement: Supplementary file 1 [file nutrients-10-01399-s001.pdf]
